# The prevalence and Covariates of Stroke in Khyber Pakhtunkhwa; From a European Perspective

**DOI:** 10.12669/pjms.37.1.3815

**Published:** 2021

**Authors:** Sahrai Saeed, Marijana Tadic, Jukka Putaala

**Affiliations:** 1 Sahrai Saeed MD, PhD, Department of Heart Disease, Haukeland University Hospital, Bergen, Norway; 2 Marijana Tadic MD, PhD, University Hospital “Dr. Dragisa Misovic - Dedinje” Department of Cardiology, Heroja Milana Tepica 1, 11000 Belgrade, Serbia; 3 Jukka Putaala MD, PhD. Neurology, Helsinki University Hospital and University of Helsinki, Helsinki, Finland

Ischemic stroke is a major public health problem and one of the leading causes of death worldwide.^1^ The risk of stroke increases with age.^2^ Although the majority of stroke patients are elderly, up to one third of first-ever strokes occur in patients <65 years, and 10-14% <55 years.^2^,^3^ Mortality rates in younger stroke patients are generally lower than older patients, but still much higher than in general population.^4^ Strokes have especially devastating implications if occur early in life given the long life expectancy during a demanding period of time in which they start to form families and make decisive career moves. Hence, stroke is a life-changing event that affect not only the person suffered, but also the entire family, as well as other caregivers. Furthermore, stroke survivors are at significantly higher risk of having recurrent vascular events.^5^ Therefore, early recognition and modification of underlying cardiovascular risk factors such as hypertension, diabetes, obesity, metabolic syndrome and smoking is crucial.^6^ We have recently highlighted the fundamental differences in the cardiovascular risk assessment of people from South Asia with particular focus on India, Pakistan and Bangladesh.^7^ These patients are often younger but have higher prevalence of pre-diabetes (insulin resistance), diabetes, abdominal obesity and an atherogenic type dyslipidemia (low HDL cholesterol and high triglycerides), despite lower total cholesterol and blood pressure (BP) levels compared with White Europeans. There are several risk stratification tools such as Framingham Risk Score, the European SCORE, and other North European risk tools such as NORRISK (Norwegian Risk) and FINRISK (Finland Cardiovascular Risk Study) to evaluate the risk of recurrent cerebrovascular event or myocardial infarction. However, most of these risk stratification tools are validated within the White European/American populations and may underestimate the risk of cardiovascular event in South Asian population.^7^

Overall, limited information exists on the prevalence, risk factors and the extent of subclinical cardiovascular disease in South Asian stroke patients, who are often younger than stroke patients of other regions.^8^ An important contribution to the current literature on the topic was the study of Sherin et al. on the prevalence and risk factors of stroke in Khyber-Pakhtunkhwa (KP), which was published in a recent edition of the Journal.^9^ A total of 15724 randomly selected households with 22500 participants (mean age 42±12.6 years, 51.4% females) were included. The prevalence of stroke was 1.25% (n=271) with a male-female ratio 1.02:1. The prevalence of obesity/overweight was 39%, hypertension 22%, smoking 7% and known diabetes 6%. Further, in their logistic regression models, age >60 years, urban area, unemployment and lower formal education were the predictors of stroke. Of note, in their study around two-third of stroke patients were less than fifty years of age.^9^ However, hypertension and diabetes, which are frequently observed in stroke patients, were not significant predictors for stroke. The true prevalence of these two conditions may be underestimated, as the information gathered was based upon formal interviews and standard BP measurements and fasting blood sugar and glycosylated hemoglobin (HbA1c) measurement were not included in the study design, which should be considered as a limitation. According to the current international guidelines on the management of systemic hypertension recommend, clinic/office BP should be measured after five minutes rest in the sitting position using a correctly sized cuff in the individual participant. BP is measured in triplets with 1-minute intervals between the measurements and the average of the two last BP measurements is taken as the clinic/office BP.^10^ Furthermore, in patients with a prevalent stroke, even in the presence of normal clinic BP, a 24-hour ambulatory BP measurement should be performed to identify: the subtypes of hypertension (Fig.1), quantify the night-time BP decline (as up to one-third of normotensive stroke patients may show a non-dipping BP pattern; i.e. <10% decline at night-time), detect daily life hypotensive episodes, as well as assess BP control in treated hypertensive patients.

**Fig.1:**
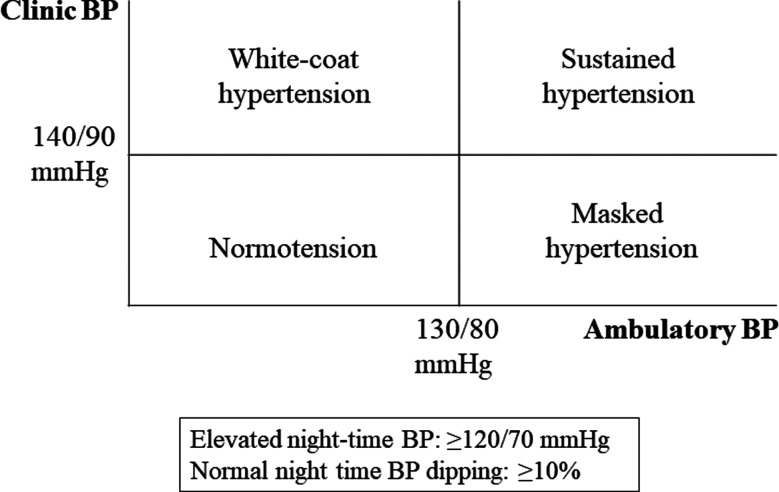
Classification of BB status according to office and ambulatory BP values. BP, blood pressure.

The second limitation, as also acknowledged by the authors, was the diagnosis of stroke *per se*. Stroke was diagnosed by the healthcare workers and not documented by neurovascular imaging (CT/MR). Assessment by a physician/neurologist and use of neurovascular imaging could also provide information, not only on differentiating hemorrhagic stroke from ischemic stroke, but also on the subtypes of ischemic stroke: large artery atherosclerosis, cardioembolic infarct, small vessel occlusion, stroke of other determined etiology, or stroke of undetermined etiology.^11^ Most importantly, subclinical brain damage (white matter lesions, lacunar infarcts, microbleeds) due to hypertension can be revealed by MR.

## Future perspectives

The study of Sherin et al.^9^ is the first largest population based study in the North West Pakistan exploring the prevalence and risk factors of stroke, and the results are translatable in the region. The database may also have the potential to provide the true prevalence and covariates of metabolic syndrome in this population. Future stroke research studies in the region should include standard clinic BP and out-of-clinic BP (either home or 24-hour ambulatory BP) measurements for patients who are apparently normotensive by clinic BP to detect masked hypertension. Further, if available, arterial stiffness derived from a pulse wave velocity should be performed at those high risk patients who show sign of hypertension-induced target organ damage, for instance left ventricular hypertrophy by echo or ECG, increased carotid intima-media thickness (>0.9 mm) or plaque by carotid ultrasound, chronic kidney disease with a eGFR <60 ml/min/1.73 m^2^, microalbuminuria (30-300 mg/24-hour, or albumin-creatinine ratio 30-300 mg/g; 3.4-34 mg/mmol, preferentially on morning spot urine), or sign of hypertensive retinopathy (hemorrhages/exudates, papilloedema). In addition to optimal treatment of diabetes and hypertension, and statins, targeted lifestyle interventions such as training and weight loss, which have shown beneficial effects in mitigating the risk of diabetes by increasing insulin sensitivity and improving glycaemic control as well as reducing BP. This is particularly important for both primary and secondary prevention of stroke at younger ages as studied by Sherin et al.
